# Carrier-doping as a tool to probe the electronic structure and multi-carrier recombination dynamics in heterostructured colloidal nanocrystals†
†Electronic supplementary information (ESI) available: Additional transient absorption spectra and kinetics, fitting procedures and fitting parameters. See DOI: 10.1039/c8sc01926f


**DOI:** 10.1039/c8sc01926f

**Published:** 2018-08-01

**Authors:** Tao Ding, Guijie Liang, Junhui Wang, Kaifeng Wu

**Affiliations:** a State Key Laboratory of Molecular Reaction Dynamics , Collaborative Innovation Center of Chemistry for Energy Materials (iChEM) , Dalian Institute of Chemical Physics , Chinese Academy of Sciences , Dalian , China 116023 . Email: kwu@dicp.ac.cn; b Hubei Key Laboratory of Low Dimensional Optoelectronic Materials and Devices , Hubei University of Arts and Science , Xiangyang , Hubei 441053 , China

## Abstract

Heterostructured colloidal nanocrystals, such as core/shells and dot-in-rods, enable new spectral and dynamic properties otherwise unachievable with single-component nanocrystals or quantum dots (QDs). For example, the electron and hole wavefunctions can be engineered such that they are either both confined in the same domain or (partially) separated over different domains in the heterostructures, which are the so-called type I or (quasi-) type II localization regimes, respectively. A critical factor dictating the carrier localization regime is the band alignment or electronic structure of the heterostructure, which, however, is difficult to measure and hence is often ambiguous. In this work, using CdSe@CdS dot-in-rods (DIRs) as a model system, we show that band edge carrier-doping is a simple-yet-powerful tool to probe the electronic structure of heterostructures. By doping an electron into the CdSe core and then observing whether the doped electron bleaches band edge absorption of only the core or those of both the core and shell, we can easily differentiate the type I and quasi-type II structures. A systematic study of DIRs with various dimensions shows that the extent of electron wavefunction delocalization can be tuned by the core sizes and rod diameters. Comparison with the electronic structure determined from transient absorption measurements also reveals the important role of electron–hole binding in affecting the delocalization of electron wavefunction. In addition to probing the electronic structure, the doped electron allows for studying multi-carrier recombination dynamics in these heterostructures which plays a vital role in their many optical and optoelectronic applications. Specifically, by comparing the band edge exciton recombination kinetics of the doped and neutral DIRs, we can extract the negative trion lifetime, which can be further used to derive the positive trion lifetime when combined with biexciton lifetime measurements. These lifetimes also depend sensitively on the core sizes and rod diameters of the DIRs.

## Abstract 2

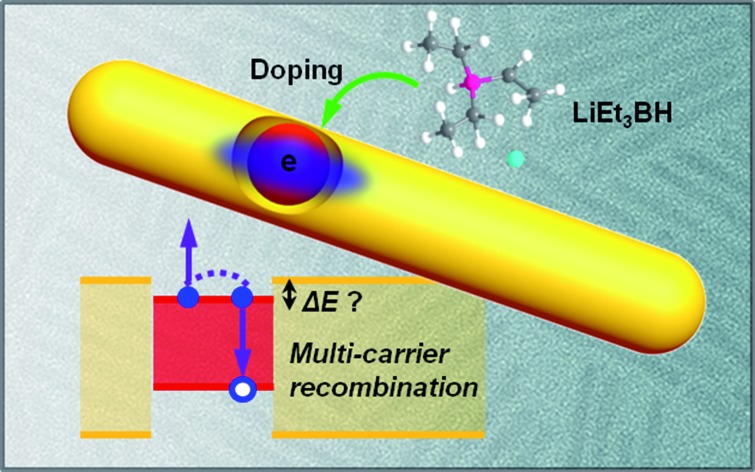
Carrier-doping enables a simple-yet-powerful tool for determination of the electronic structure and multi-carrier recombination dynamics of CdSe@CdS dot-in-rods.

## Introduction

Decades of development in colloidal nanocrystal (NC) chemistry has now allowed for the synthesis of not only monocomponent nanocrystals with well-controlled size and shape but also heterostructured nanocrystals (hetero-NCs) comprising two or more material components.^1^–^8^ These hetero-NCs can have various dimensionalities, such as zero-dimensional (0-D) core/shell quantum dots (QDs),^3^,^5^,^9^–^11^ 1-D linear or core/shell hetero-nanorods (NRs),^7^,^12^–^15^ and 2-D core/crown nanoplatelets (NPLs);^16^–^20^ they can even be of mixed dimensionality, such as dot-in-rods (DIRs)^21^–^24^ and dot-in-platelets (DIPLs).^25^ These heterostructures enable new spectral and dynamic properties that are otherwise very difficult to achieve using single-component NCs.^2^,^26^,^27^ Arguably, almost all the prospective applications of NCs, ranging from photocatalysis,^27^ light-emitting diodes (LEDs),^28^lasers,^26^ luminescent solar concentrators (LSCs),^29^ to bio-labeling and tagging,^30^ require the use of heterostructures.

A key parameter enabling the spectral and dynamic properties for the aforementioned applications is the band alignment or electronic structure of the hetero-NCs. Depending on the specific choices of materials and their sizes, the alignment of the conduction (CB) and valence (VB) band edges of the components in the hetero-NCs can be either so-called type I, in which both the CB and VB of one component are confined within those of the other,^3^,^4^ type II which features a staggered alignment of band edges,^10^,^31^ or quasi-type II, in which either the CB or VB band edges are similar in the component materials.^32^,^33^ In the following, we use the term “electronic structure” rather than “band alignment” because one may argue that band alignment is a more suitable term for bulk materials only. The electronic structure of hetero-NCs determines the wavefunction distributions of electrons and holes in the excited states, which are directly linked to the carrier (de)localization behaviors.

Albeit being very important for prospective applications of hetero-NCs, the electronic structure is difficult to determine. One of the typical examples is the CdSe@CdS DIRs with a CdSe QD embedded in a CdS rod.^21^–^23^,^29^,^32^,^34^–^40^ Because of a large VB offset (>0.45 eV) and a relatively smaller CB offset (<0.3 eV) between bulk wurtzite CdSe and CdS^21^,^36^ and because of the quantum confinement effect primarily for electrons in CdSe and CdS, the electronic structure of CdSe@CdS DIRs can be either type I with both the electron and hole confined in CdSe or quasi-type II with the hole confined in CdSe but the electron delocalized among CdSe and CdS, depending sensitively on the size of the CdSe QD and the diameter of the CdS rod.

Over the years, the electronic structure of this system has been studied using various techniques, including scanning tunneling spectroscopy,^36^ transient absorption and fluorescence spectroscopy.^29^,^32^,^35^,^39^,^40^ In principle, only the scanning tunneling technique probes the intrinsic, “single-particle” electronic structure, as various optical techniques probe not single-particle but excitonic states which contain the contribution from electron–hole coulombic interactions. Recent studies have shown that in these low-dimensional materials, electron–hole binding energy can be strong enough to significantly modify the wavefunction distributions of electrons and/or holes.^41^–^45^ Recognizing the complexity of scanning tunneling measurements, we have yet to develop a relatively simple technique for the facile determination of the single-particle electronic structure of hetero-NCs. One such example is the spectro-electrochemistry method in which an electric bias is applied to inject charges into NCs.^46^

In addition to the electronic structure, another fundamental process that is crucial for the application of hetero-NCs is the multi-carrier recombination process.^47^,^48^ It is now established that in semiconductor NCs, due to quantum confinement effects, multi-carrier recombination is dominated by nonradiative Auger decay whereby the electron–hole recombination energy is not released as a photon but instead transferred to a third charge.^49^,^50^ This has been considered the key obstacle to QD-lasers^51^–^54^ as the optical gain of QDs intrinsically relies on multi-exciton states which can quickly decay *via* Auger recombination.^26^,^54^ For this reason, multi-exciton recombination dynamics has been extensively studied for various single-component and hetero-NCs.^50^,^55^,^56^ Compared to these neutral multi-excitons, charged excitons have received less attention. However, recent studies have revealed that charged excitons could be the primary excited states for NC-devices under the operating conditions as NCs are often unintentionally charged due to an imbalance between electron and hole injection (or extraction) rates in LEDs (or solar cells and photocatalytic systems).^57^,^58^

**Scheme 1:**
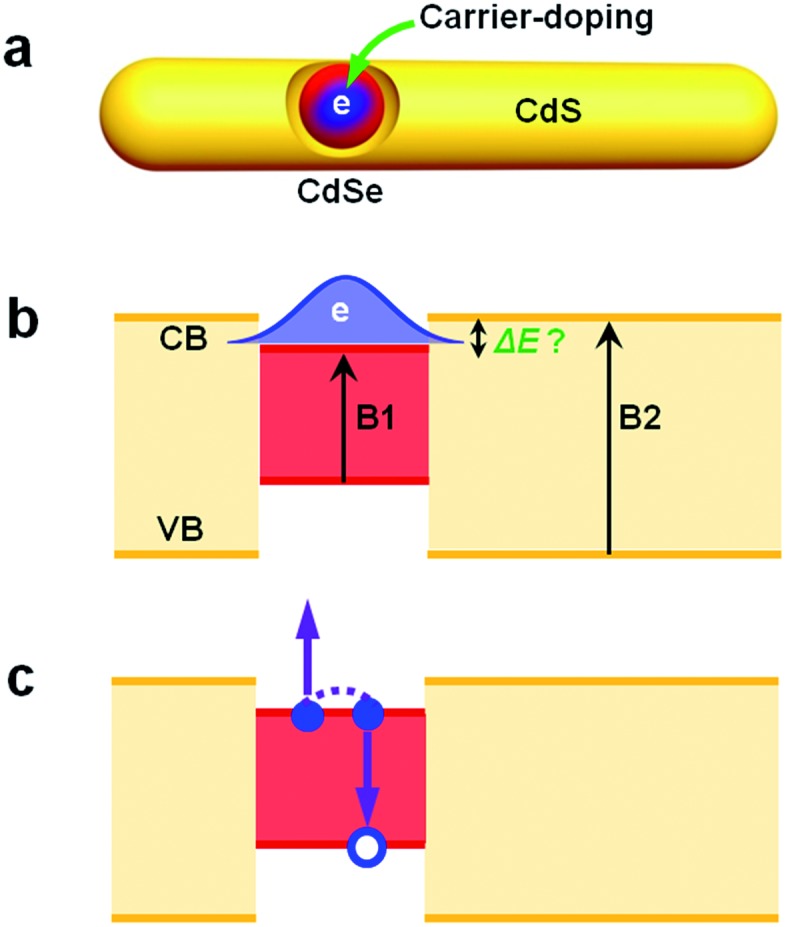
Carrier-doping to probe the electronic structure and multi-carrier dynamics in CdSe@CdS dot-in-rods (DIRs). (a) Schematic illustration of injecting an electron into the CdSe core *via* a photochemical doping method. (b) The doped electron can be used to differentiate type I and quasi-type II electronic structures, which correspond to a conduction band (CB) offset Δ*E* of 0 and >0, respectively, by observing whether the electron blocks only the B1 transition or both B1 and B2 transitions. (c) The doped electrons also allow for studying the multi-carrier recombination dynamics of negative trions (X^–^) which can be further used to measure positive trions (X^+^) when combined with biexciton recombination measurements.

In this paper, we report carrier-doping as a simple yet powerful tool to simultaneously probe the single-particle electronic structure and multi-carrier recombination dynamics of hetero-NCs. Using CdSe@CdS DIRs as a model system, we show that by doping an electron (n-doping) into the CdSe core (Scheme 1a) and then observing the occupation behavior of the doped electrons (Scheme 1b), we can readily differentiate type I and quasi-type II electronic structures. The measured results can be compared with those from optical techniques, such as transient absorption, to uncover the role of the hole in affecting the (de)localizing behavior of the electron. By measuring the difference between the band edge exciton recombination kinetics of the n-doped and neutral DIRs, we can also extract trion (X^–^) recombination kinetics which is dominated by Auger decay (Scheme 1c).

## Results and discussion

### Morphologies and optical properties of CdSe@CdS DIRs

**Fig. 1:**
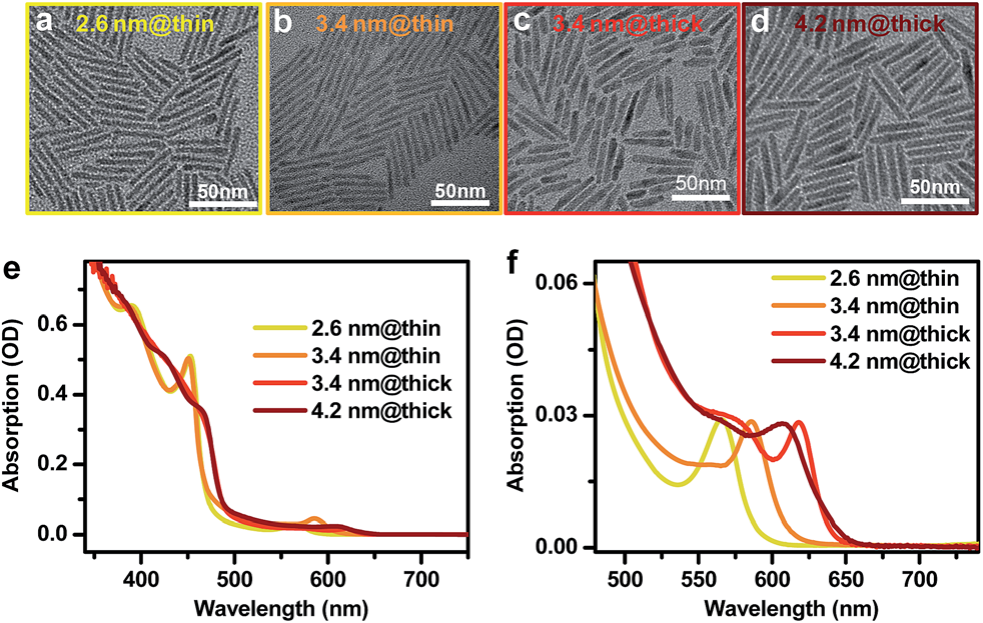
Morphologies and optical properties of CdSe@CdS DIRs with various dimensions. (a–d) Representative TEM images of DIRs with (a) a 2.6nm-diameter core embedded in a thin rod, (b) a 3.4nm-diameter core embedded in a thin rod, (c) a 3.4nm-diameter core embedded in a thick rod, and (d) a 4.2nm-diameter core embedded in a thick rod, which are labeled as 2.6 nm@thin, 3.4 nm@thin, 3.4 nm@thick, and 4.2 nm@thick, respectively. (e) Absorption spectra of 2.6 nm@thin (yellow), 3.4 nm@thin (orange), 3.4 nm@thick (red), and 4.2 nm@thick (brown) DIRs. (f) Enlarged view of the CdSe core absorption in (e).

**Fig. 2:**
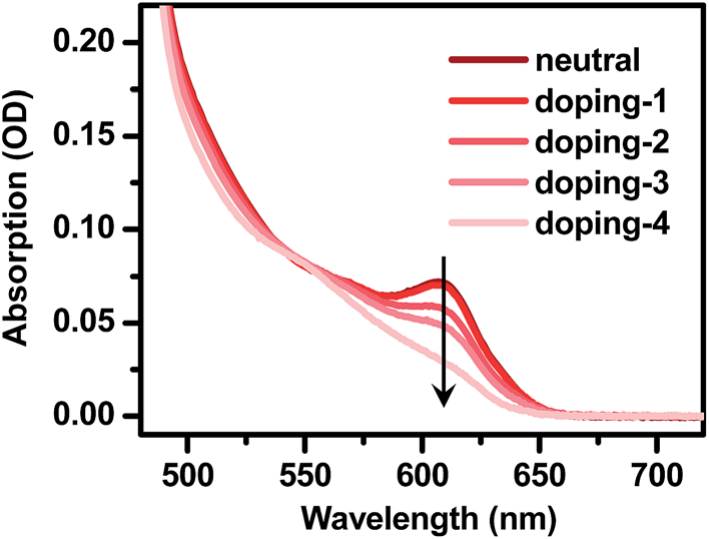
Electron-doping of CdSe@CdS DIRs. Absorption spectra of 4.2 nm@thick DIRs showing progressively bleached core exciton absorption with increasing amount of injected electrons.

CdSe@CdS DIRs with various dimensions were synthesized using literature methods with slight modifications in the reaction temperature and time.^16^,^52^,^59^,^60^ Details of the synthesis can be found in the ESI.† We used CdSe QDs of three different diameters (2.6 nm, 3.4 nm, and 4.2 nm) as the seeds for growth of CdS rods. Four DIR samples were made from these seeds, which are labeled as 2.6 nm@thin, 3.4 nm@thin, 3.4 nm@thick, and 4.2 nm@thick based on the seed and rod diameters. “Thin” and “thick” refer to the diameter of the CdS rod being either thin (with a strong quantum confinement effect) or thick (with weak confinement). Representative transmission electron microscopy (TEM) images of these DIRs are shown in Fig. 1a–d. Statistics on the dimensions shows that the 2.6 nm@thin, 3.4 nm@thin, 3.4 nm@thick, and 4.2 nm@thick samples have average diameters (lengths) of 3.4 ± 0.6 nm (40.0 ± 4.0 nm), 3.6 ± 0.5 nm (44.3 ± 3.4 nm), 5.7 ± 0.8 nm (36.6 ± 8.6 nm), and 5.6 ± 0.9 nm (40.5 ± 3.5 nm), respectively; see the distribution histograms in Fig. S1 in the ESI.† Therefore, within the standard deviations, the lengths of all four samples are controlled to be similar and are well beyond the Bohr exciton diameter of CdS (*d*_B_ ∼ 5.6 nm (ref. 61)). The diameters of the two thin DIRs are smaller than *d*_B_, and hence the CdS rods for these two samples are strongly confined; in contrast, the diameters of the two thick DIRs are close to *d*_B_, and the CdS rods are only weakly confined. Indeed, the absorption spectra of these samples in Fig. 2e show that the two thin samples exhibit well-resolved, quantum-confined CdS excitonic bands at ∼450 nm, whereas the two thick samples exhibit broad absorption features with onset at ∼490 nm. In addition to the dominant rod absorption features, an enlarged view of the absorption spectra in the long wavelength range reveals the excitonic bands of the CdSe seeds (Fig. 1f). These absorption features are significantly red-shifted as compared to those of the original CdSe QDs before rod coating (Fig. S2†), indicative of wavefunction, primarily electron wavefunction, leakage into the rods. The red-shifts are 162 meV, 82 meV, 192 meV, and 73 meV for 2.6 nm@thin, 3.4 nm@thin, 3.4 nm@thick, and 4.2 nm@thick, respectively. A larger red-shift corresponds to stronger electron wavefunction delocalization.

The photoluminescence (PL) spectra of these DIRs are shown in Fig. S3a,† with PL quantum yields (QYs) typically in the range of 40–50% (excited at 450 nm). Based on a previous report,^62^ a major PL efficiency loss of CdSe@CdS DIRs when excited at the CdS rod is the ultrafast hole trapping on rod surfaces that competes with exciton localization from the rod to the seed; the localization efficiency is ∼60% for a 40 nm-long rod which is similar to but slightly higher than the PL QYs of our samples, indicating that electron–hole recombination inside the CdSe core is dominated by radiative decay only. Time-resolved PL decay measurements show that all the DIRs exhibit near single-exponential decays (Fig. S3b†), with time constants of 8.13± 0.01 ns, 8.03 ± 0.01 ns, 12.9 ± 0.02 ns, and 12.1 ± 0.02 ns for 2.6 nm@thin, 3.4 nm@thin, 3.4 nm@thick, and 4.2 nm@thick, respectively.

### Photochemical electron-doping of CdSe@CdS DIRs

In order to dope an electron into the CdSe@CdS DIRs, we adopt a recently developed photochemical doping method.^63^ In this method, the photogenerated holes in the VB of CdSe@CdS DIRs are scavenged by a reducing reagent, lithium triethylborohydride (LiEt_3_BH), leaving behind photogenerated electrons in the CB of DIRs. A key to enabling permanent electron accumulation in the CB is that upon accepting holes, LiEt_3_BH is rapidly decomposed, which prevents charge recombination between electrons in DIRs and holes in LiEt_3_BH. The details of photochemical doping experiments can be found in the ESI.† Note that we choose to dope electrons instead of holes because for the II–VI group NCs electrons are much more effective in bleaching the band-edge absorption than holes, which would expedite the study of doped electrons using optical spectroscopy,^64^,^65^ and also because, for our specific case of CdSe@CdS DIRs, it is the electron wavefunction that depends sensitively on the electronic structure.

As illustrated in Fig. 2 using the 4.2 nm@thick sample as an example, the addition of LiEt_3_BH to deoxygenated DIR solution under an inert atmosphere in a N_2_-filled glove box and exposure to UV light caused progressive bleaching of the absorption bands of the CdSe core. This is indicative of electron injection into the 1S_e_ level of the CdSe core, which partially blocks the transition into this level due to the state-filling effect.^63^ Exposure to air immediately removes the doped electrons as indicated by the rapid recovery of the absorption spectrum; thus all the measurements of n-doped QDs were performed under strictly air-free conditions.

### Electronic structure of CdSe@CdS DIRs

As briefly discussed in the Introduction, the doped electron should allow for facile differentiation between type I and quasi-type II electronic structures by observing whether the doped electron bleaches band edge absorption of only the core or those of both the core and shell. We applied the photochemical n-doping to all DIR samples. Note that the doping level was controlled to be low such that the bleach of the 1S excitonic band of the CdSe core was within 20%, which ensures that DIRs in the ensemble are dominated by either undoped species or species doped with one electron.

**Fig. 3:**
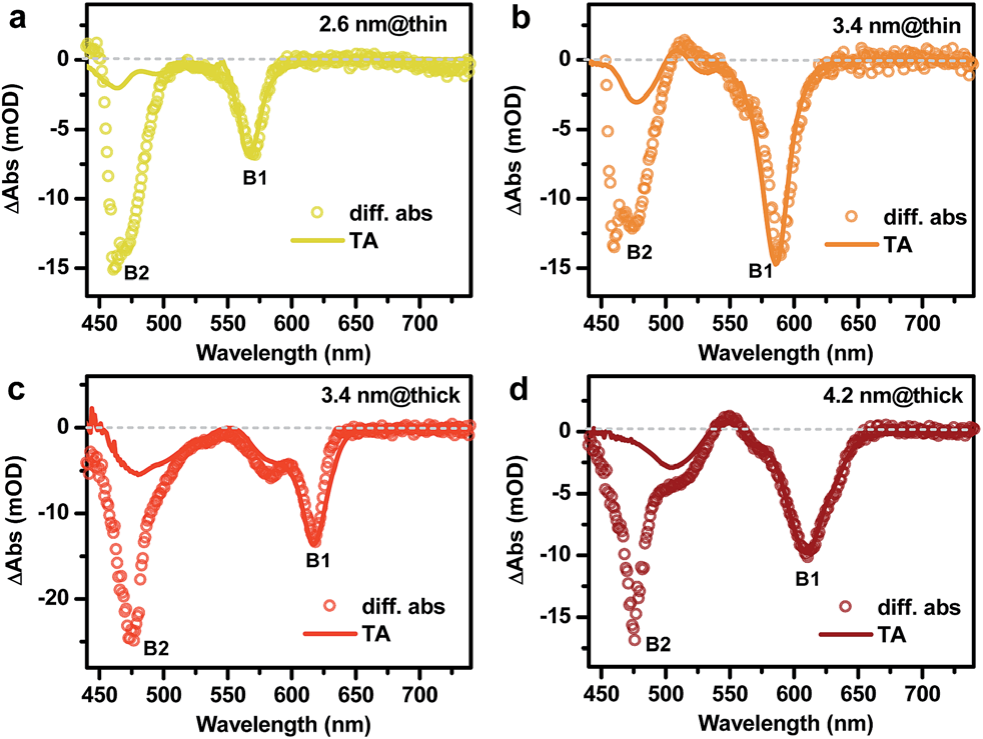
Difference spectra between the absorption spectra of n-doped and neutral DIRs (open circles) and transient absorption (TA) spectra of corresponding NR samples (solid lines). (a) 2.6 nm@thin DIRs. (b) 3.4 nm@thin DIRs. (c) 3.4 nm@thick DIRs. (d) 4.2 nm@thick DIRs. The TA spectra are averaged between 1 and 3 ps following near-resonant excitation of the core absorption bands. B1 and B2 spectral features are indicated.

According to the absorption spectra in Fig.S4,† the doping procedure leads to bleaching of the CdSe core absorption bands but its effect on the absorption of CdS rods is not as obvious. However, by taking a difference between the same doped sample before (n-doped) and after (neutral) exposure to air, bleaching of both the CdSe core (B1) and the CdS rod (B2) absorption can be observed, as shown in Fig. 3. For the 2.6 nm@thin DIR, the amplitude of the B2 bleach is ∼2.2 times that of the B1 bleach (open circles in Fig. 3a), indicative of strong leakage of electron wavefunction from the CdSe core to the CdS rod. This is consistent with the strong red-shift (∼162 meV) of the core absorption band in this DIR compared to the original CdSe QD absorption. In contrast, the amplitude of the B2 bleach in the 3.4 nm@thin DIR (open circles in Fig. 3b) is similar to that of the B1 bleach and is much lower than the relative amplitude of the B2 bleach in the 2.6 nm@thin DIR, again consistent with the weak red-shift (∼82 meV) of the core absorption band in this DIR compared to the original CdSe QD absorption. Considering that these two samples have similar rod diameters, the comparison between them shows that with a fixed rod diameter the extent of electron wavefunction delocalization can be tuned by the core size, with smaller cores favoring stronger delocalization.

For the 3.4 nm@thick DIR (Fig. 3c), the amplitude of the B2 bleach is ∼2-fold higher than that of the B1 bleach. The comparison of this result with that of 3.4 nm@thin DIR demonstrates that with a fixed core size, the rod diameter can also affect the extent of electron wavefunction delocalization, with larger rod diameters favoring stronger delocalization. At last, for the 4.6 nm@thick DIR (Fig. 3c), the amplitude of the B2 bleach is ∼1.7-fold that of the B1 bleach. The comparison between 3.4 nm@thick and 4.6 nm@thick DIRs also demonstrates that when the rod diameter is relatively fixed the extent of electron wavefunction delocalization can be tuned by the core size. Thus, through the comparisons between these four DIRs, we clearly illustrate how the wavefunction delocalization behavior can be engineered through tuning the core size and rod diameter. It is also important to note that for all the DIRs studied here, within the framework of the single-particle picture, the electronic structure is quasi-type II. This is because the small CB offset between CdSe and CdS (<0.3 eV) is not sufficient to confine the electron motion completely in the CdSe core for the size range we studied.

It is informative to compare the above results probed by electron-doping to those measured using transient absorption (TA) spectroscopy.^35^,^39^,^45^ In these TA experiments, we selectively excited the B1 features from the core by tuning the pump photon energy to be slightly above the peak energy of the core absorption bands. The resulting TA spectra (averaged between 1 and 3 ps delay after the pump pulse; see more spectra in Fig. S5†) for these DIRs are plotted in Fig. 3 (solid lines) for a direct comparison with their corresponding doping-induced difference spectra. For all the samples, the spectral shapes of B1 bleaches created by n-doping and by TA are similar, as both arise from the state filling effect of the 1S_e_ electrons in the core. To compare the spectral shapes of B2 bleaches, we normalize the doping-induced difference spectra and TA spectra at the B1 bleaches. It can be seen that for all the samples, the amplitudes of the B2 bleaches on the TA spectra (*A*_B2,TA_) are much lower than those on the doping-induced difference spectra (*A*_B2,doping_). This difference results from different natures of the electrons created by these two methods. The doped electron is an independent single-particle whereas the electron created in TA is bound to a hole by the coulomb potential. This potential acts as an additional barrier for electron delocalization, which accounts for the lower *A*_B2,TA_ than *A*_B2,doping_. Specifically, the ratios between *A*_B2,TA_ and *A*_B2,doping_ are ∼14%, ∼24%, and ∼25% for 2.6 nm@thin, 3.4 nm@thin and 3.4 nm@thick DIRs, respectively. The lower ratio in 2.6 nm-seeded DIRs than that in 3.4 nm-seeded ones likely reflects a stronger electron–hole binding energy in the former, which is consistent with the smaller size of the core. Particularly noteworthy is that *A*_B2,TA_ is almost zero for the 4.2 nm@thick DIR and the weak signal at this wavelength is merely a tail from the absorption band at ∼500 nm. This observation indicates that the 4.2 nm@thick DIR can be treated as a type I structure from TA measurements, whereas the electron-doping reveals it as a quasi-type II structure from the single-particle point of view. This apparent conflict emphasizes the important role of electron–hole binding in affecting the delocalization behavior of the electron.

### Multi-carrier recombination dynamics in CdSe@CdS DIRs

**Fig. 4:**
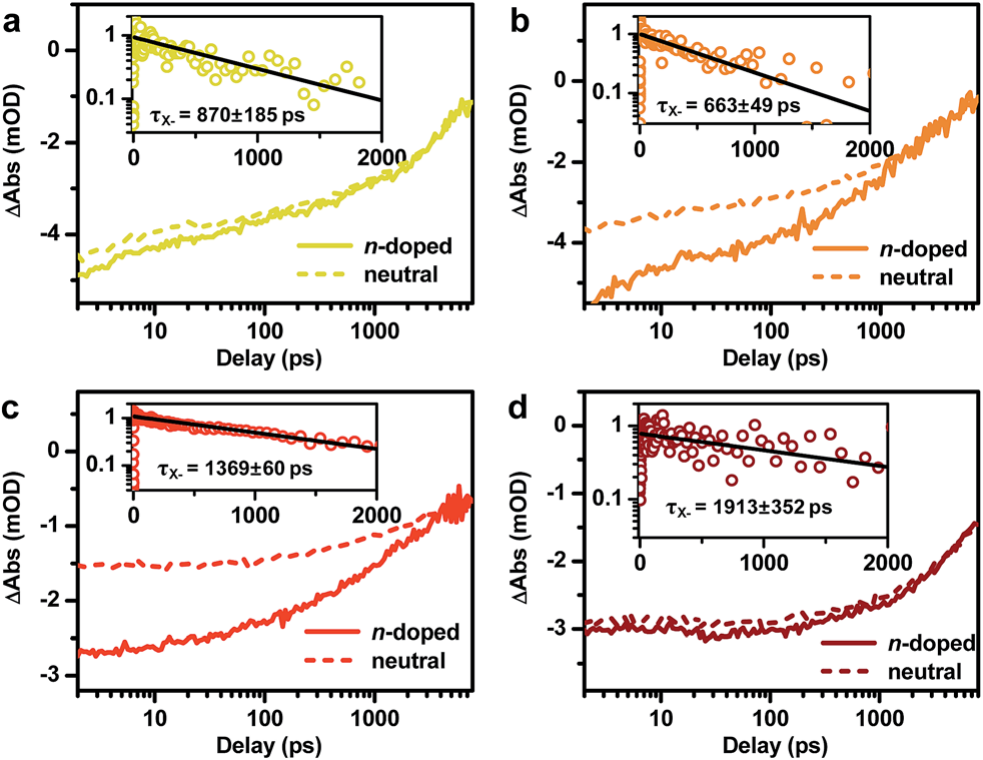
Negative trion lifetime of DIRs. TA kinetics probed at the B1 feature for (a) 2.6 nm@thin, (b) 3.4 nm@thin, (c) 3.4 nm@thick, and (d) 4.2 nm@thick DIRs. The kinetic traces for n-doped and neutral samples are normalized to the long-lived components and are shown as solid and dashed lines, respectively. The subtractions between them generate the recombination kinetics of negative trions (open circles) and the black solid lines are single-exponential fits to them (insets).

In addition to probing the electronic structure, the doped electron offers an opportunity to study multi-carrier recombination dynamics in charged excitonic states. Specifically, the excitation of an n-doped NC should create a negative trion (X^–^) which decays faster due to its higher rates of both radiative and Auger recombinations.^66^–^69^ Here we measure the X^–^ recombination dynamics of DIRs using TA spectroscopy. The TA spectra of n-doped and neutral DIRs excited by 500 nm pulses are provided in Fig. S6.† In these measurements, the average initial exciton number ( In these measurements, the average initial exciton number (〈*N*〉) was controlled to be low (<0.1) such that trion states instead of more complex states such as charged biexcitons are measured. As shown in ) was controlled to be low (<0.1) such that trion states instead of more complex states such as charged biexcitons are measured. As shown in Fig. 4, the kinetics of n-doped and neutral DIRs probed at the B1 feature can be re-scaled such as to match their long-lived components which correspond to the subset of undoped DIRs in the n-doped sample. A subtraction between the re-scaled TA kinetics can remove the contribution from undoped DIRs and generate kinetics purely due to X^–^ (Fig. 4 insets). For all the samples, the kinetics can be fitted to single-exponential decay functions with time constants of 870 ± 185 ps, 663 ± 49 ps, 1369 ± 60 ps, and 1913 ± 352 ps for 2.6 nm@thin, 3.4 nm@thin, 3.4 nm@thick, and 4.2 nm@thick DIRs, respectively (Table 1). These time constants are assigned to the X^–^ lifetimes (*τ*_X^–^_) of DIRs, which should contain contributions from both radiative and nonradiative Auger recombinations. According to previous reports,^48^,^70^–^72^ the radiative lifetime of X^–^ (*τ*_X^–^,r_) is ∼1/1.7 that of single excitons (*τ*_X_) for CdSe@CdS systems, with *τ*_X_ already determined from time-resolved PL measurements (Fig. S3b†). We can thus estimate the emission QYs of X^–^ in DIRs relative to single excitons using:*Φ*_X^–^_ = *τ*_X^–^_/*τ*_X^–^,r_ (Table 1). The QYs are in the range of 14% to 24% and are relatively higher in DIRs with more strongly delocalized electron wavefunctions (2.6 nm@thin and 3.4 nm@thick) or with large volume (4.2 nm@thick). These QYs might explain the “gray” states observed in single-particle PL experiments of CdSe@CdS NRs^73^ as the photocharged species, usually being X^–^ for CdSe@CdS systems,^70^ are reasonably bright rather than completely dark.

**Table 1:** Lifetimes and emission yields (relative to single excitons) of multi-carrier states

	*τ*_X^–^_ (ps)	*τ*_X^+^_ (ps)	*τ*_XX_ (ps)	*Φ*_X^–^_ (%)	*Φ*_X^+^_ (%)	*Φ*_XX_ (%)
2.6 nm@thin	870	122	57.8	16.0	3.02	2.84
3.4 nm@thin	663	189	83.9	12.4	4.71	4.18
3.4 nm@thick	1369	744	303	15.9	11.5	9.40
4.2 nm@thick	1713	767	326	21.2	12.7	10.8

Although the direct creation and measurement of positive trions (X^+^) is difficult for II–VI group QDs,^70^ they can be inferred using a well-established interdependence between X^–^, X^+^, and biexciton (XX) lifetimes.^48^,^70^,^71^ To this end, we measured the XX recombination dynamics in our DIRs. In these experiments, 500 nm pump pulses with progressively increasing energy densities were used to excite the samples. We use 500 nm instead of 400 nm excitation that was extensively applied in the literature for multi-exciton experiments as 400 nm excitation could also create trapped excitons on the rod surfaces due to non-unity-yield exciton localization from the rod to the core.^45^,^62^,^74^ 500 nm excitation can not only help minimize this effect, as the absorption of the rod at this wavelength is weak, but also is far enough away from the CdSe band edge to guarantee a Poissonian behavior for the statistics of photon absorption events.

**Fig. 5:**
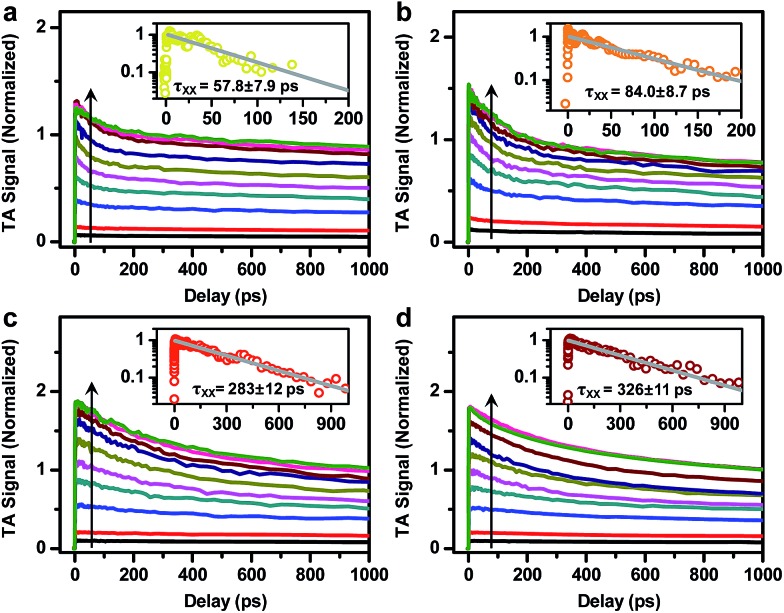
Biexciton lifetime of DIRs. TA kinetics probed at the B1 feature for (a) 2.6 nm@thin, (b) 3.4 nm@thin, (c) 3.4 nm@thick, and (d) 4.2 nm@thick DIRs measured at various excitation energy densities from 14 to 2240 μJcm^–2^ (colored solid lines). The subtractions between low-excitation-density kinetic traces generate the recombination kinetics of biexcitons (open circles) and the black solid lines are single-exponential fits to them (insets).

Fig. 5 shows the TA kinetics probed at the B1 feature for the DIRs measured at various excitation energy densities from 14 to 2240 μJcm^–2^. To avoid photocharging effects, samples were vigorously stirred during all the measurements. The complete sets of TA spectra are provided in Fig. S7–S10.† Note that the TA kinetics in Fig. 5 have been rescaled to set the saturated signal amplitudes of the slowly decaying components to 1. Fig.S11† shows the plots of the rescaled signal amplitudes as a function of the excitation densities (blue squares) and their fits to a Poisson statistics model (see the ESI† for details),^50^ from which the initial average exciton number from which the initial average exciton number 〈*N*〉 at each pump density can be calculated. The XX recombination kinetics (open circles) can then be obtained by performing a subtraction between XB kinetics with 〈 at each pump density can be calculated. The XX recombination kinetics (open circles) can then be obtained by performing a subtraction between XB kinetics with 〉 at each pump density can be calculated. The XX recombination kinetics (open circles) can then be obtained by performing a subtraction between XB kinetics with 〈*N*〉<0.5 (<0.5 (Fig. 5 insets). These kinetics can be fitted to single-exponential decay functions with time constants (*τ*_XX_) of 57.8 ± 7.9 ps, 84.0 ± 8.7 ps, 283 ±12 ps, and 326 ± 11 ps for 2.6 nm@thin, 3.4 nm@thin, 3.4 nm@thick, and 4.2 nm@thick DIRs, respectively (Table 1). According to standard statistical scaling of radiative rates,^48^*τ*_XX,r_ is 1/4 of the single exciton radiative lifetime. Using this information, the emission QYs of XX in DIRs relative to single excitons can be estimated using: *Φ*_XX_ = *τ*_XX_/*τ*_XX,r_ (Table 1). The QYs are in the range of 2.8% to 10.8%.

The emission QYs of XX are much lower than those of X^–^ because the nonradiative Auger recombination process in an XX consists of two X^–^ and two X^+^ pathways.^71^ This is the superposition principle that can be used to calculate the lifetime of X^+^ (*τ*_X^+^_) in DIRs (see the ESI and Table S1† for details). The calculated values are 122 ps, 188 ps, 740 ps, and 762 ps for 2.6 nm@thin, 3.4 nm@thin, 3.4 nm@thick, and 4.2 nm@thick DIRs, respectively (Table 1), significantly faster than the corresponding X^–^ lifetimes in these DIRs. Their emission QYs relative to single excitons (*Φ*_X^+^_) are in the range of 3.0% to 12.6%, which are much lower than those of X^–^. The asymmetries between X^+^ and X^–^ lifetimes, and between X^+^ and X^–^ emission yields have at least two reasons:^70^(1) in general, the electron and hole density of states are very different in II–VI group QDs and, as a result, Auger recombination for intraband re-excitation of the hole (which is the X^+^ pathway) is easier and thus faster than for re-excitation of the electron; (2) in CdSe@CdS structures, electron wavefunctions are more delocalized than those of holes, generating a much large effective volume factor for the electron which suppresses Auger recombination of X^–^. This observation is consistent with previous reports on the X^+^ and X^–^ lifetimes of CdSe/CdS core/shell QDs.^66^,^70^,^71^

## Conclusions

In conclusion, we have applied electron-doping as a tool to simultaneously probe the electronic structure and multi-carrier recombination dynamics of CdSe@CdS DIRs. We show that the doped electron allows for facile differentiation between type I and quasi-type II electronic structures by simply measuring the absorption spectra. A study of DIRs with various dimensions showed that the extent of electron wavefunction delocalization could be engineered through both the core sizes and rod diameters. The comparison of results from these measurements with those from transient absorption highlights the role of the hole in affecting the delocalization behavior of the electron through the coulomb potential. In particular, for one DIR sample, the “single-particle” electronic structure revealed by electron-doping experiment was quasi-type II, whereas TA measurements showed that its “excitonic” electronic structure was type I. The doped electron also offers an opportunity to probe multi-carrier recombination dynamics which is crucial for the application of these heterostructures in light emission and conversion. By measuring the difference between the TA kinetics of the doped and undoped DIRs, we extracted the negative trion (X^–^) recombination kinetics, which was further combined with biexciton (XX) measurements to derive the positive trion (X^+^) lifetime. The decay of these multi-carrier states is dominated by Auger recombination, which also depends sensitively on the core sizes and rod diameters as they affect the wavefunction distribution and effective volume factor of the DIRs.

## Conflicts of interest

There are no conflicts to declare.

## Supplementary Material

Supplementary information
