# Supplementary material for: Carrier-doping as a tool to probe the electronic structure and multi-carrier recombination dynamics in heterostructured colloidal nanocrystals
Source: Chem Sci. 2018 Aug 1;9(36):7253–60. doi: 10.1039/c8sc01926f (PMC6148752; doi:10.1039/c8sc01926f)
Supplement: Supplementary file 1 [file SC-009-C8SC01926F-s001.pdf]

Electronic Supplementary Material (ESI) for Chemical Science.
This journal is © The Royal Society of Chemistry 2018

## Electronic Supplementary Information (ESI)

# Carrier-doping as a tool to probe the electronic structure and multi-carrier recombination dynamics in heterostructured colloidal nanocrystals

Tao Ding,‡[a] Guijie Liang,‡[ab] Junhui Wang,[a] and Kaifeng Wu*[a]

[a]State Key Laboratory of Molecular Reaction Dynamics and Collaborative Innovation Center of Chemistry for Energy Materials (iChEM), Dalian Institute of Chemical Physics, Chinese Academy of Sciences, Dalian, China, 116023

[b]Hubei Key Laboratory of Low Dimensional Optoelectronic Materials and Devices, Hubei University of Arts and Science, Xiangyang, Hubei 441053, China

*Corresponding author. E-mail: kwu@dicp.ac.cn

‡T. Ding and G. Liang contributed equally to this work.

**Contents:**

Figs. S1-S11

Synthesis of CdSe@CdS DIRs

Photochemical electron-doping of DIRs

Transient absorption (TA) measurements

Calculation of lifetimes and emission yields of multi-carrier states

Table S1

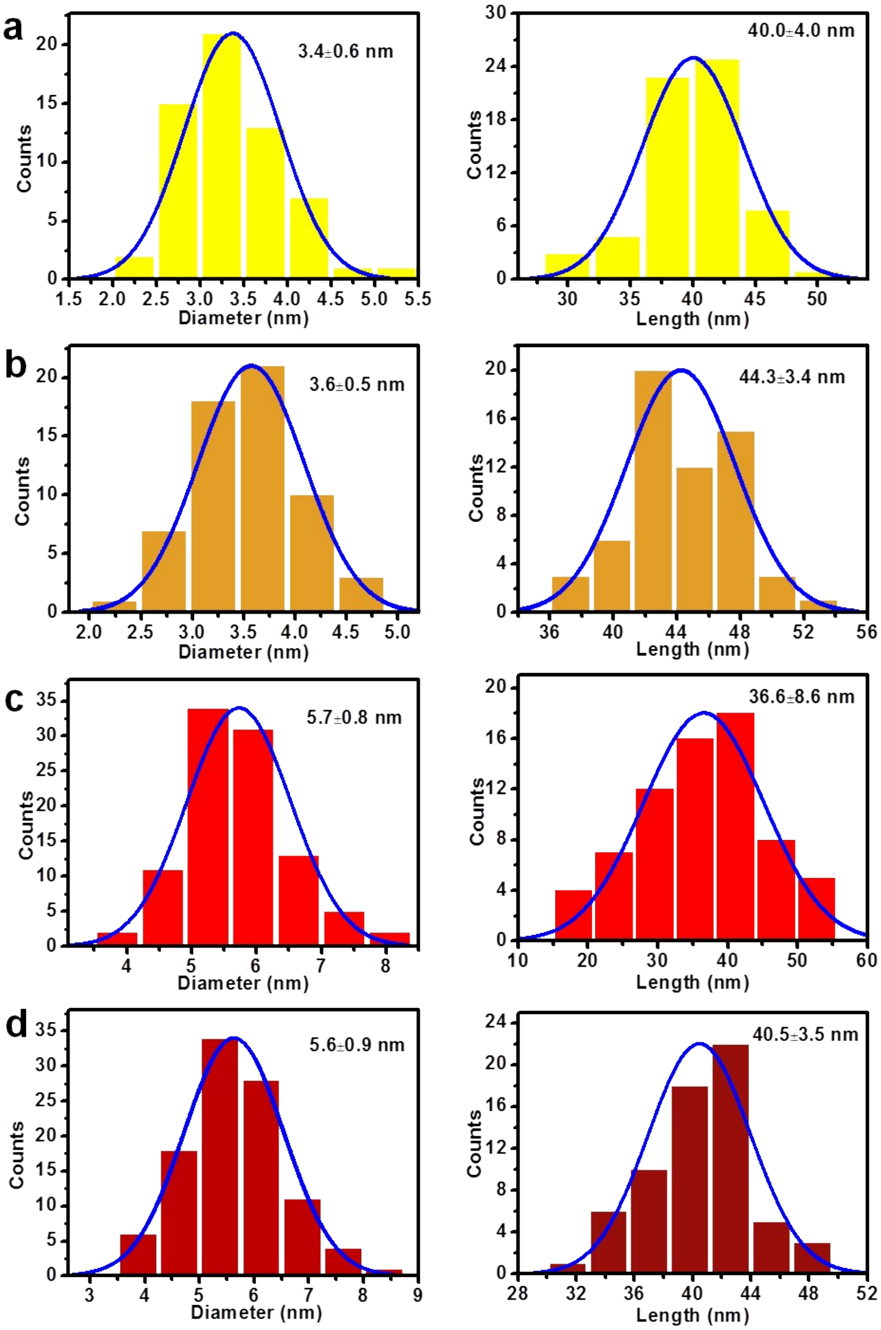


**Fig. S1** Histograms for the rod diameters (left panels) and lengths (right panels) for (a) 2.6 nm@thin DIRs, (b) 3.4 nm@thin DIRs, (c) 3.4 nm@thick DIRs, and (d) 4.2 nm@thick DIRs.

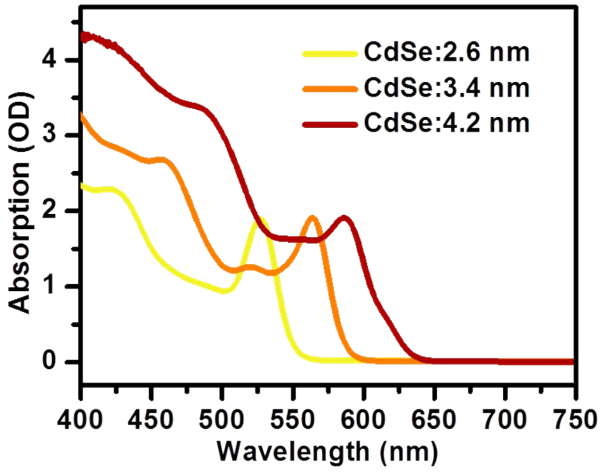


**Fig. S2** Absorption spectra of the CdSe seeds used for the growth of DIR samples.

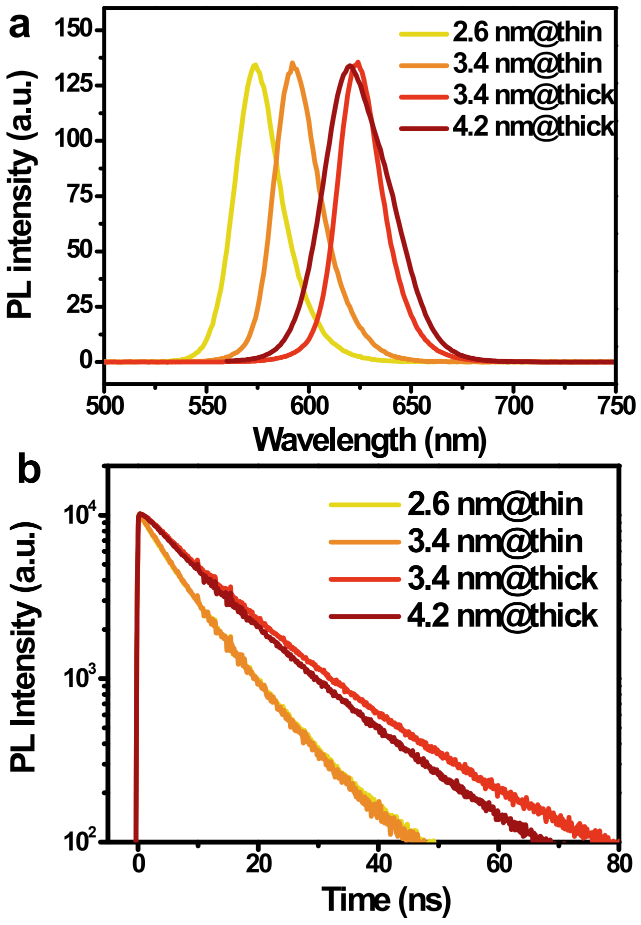


**Fig. S3** (a) PL spectra and (b) time-resolved PL kinetics of the DIR samples. The excitation wavelength is 450 nm. The PL kinetics are monitored at the peaks of the PL bands.

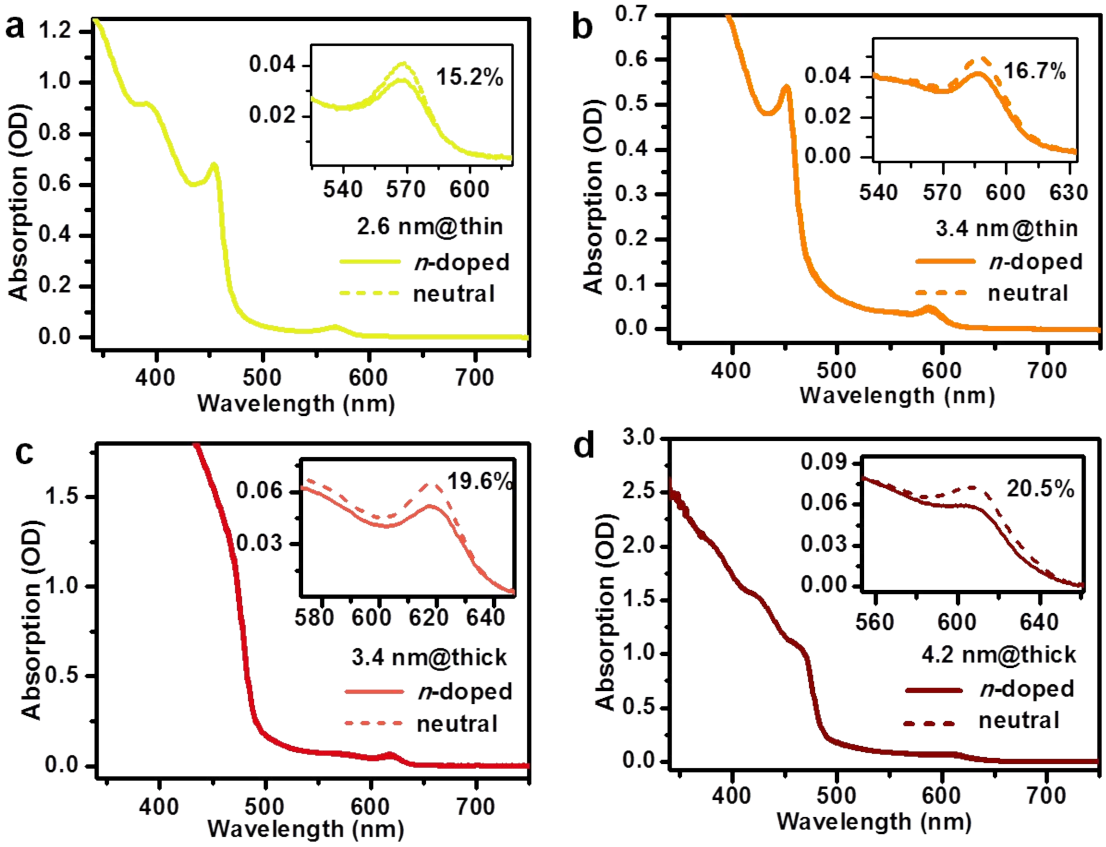


**Fig. S4** Absorption spectra of *n*-doped (solid lines) and neutralized (by exposure to the air; dashed lines) samples for (a) 2.6 nm@thin DIRs, (b) 3.4 nm@thin DIRs, (c) 3.4 nm@thick DIRs, and (d) 4.2 nm@thick DIRs. Insets show the enlarged view of the spectra near the core 1S exciton bands. The labeled numbers indicate the extents of bleaching at the 1S exciton peak.

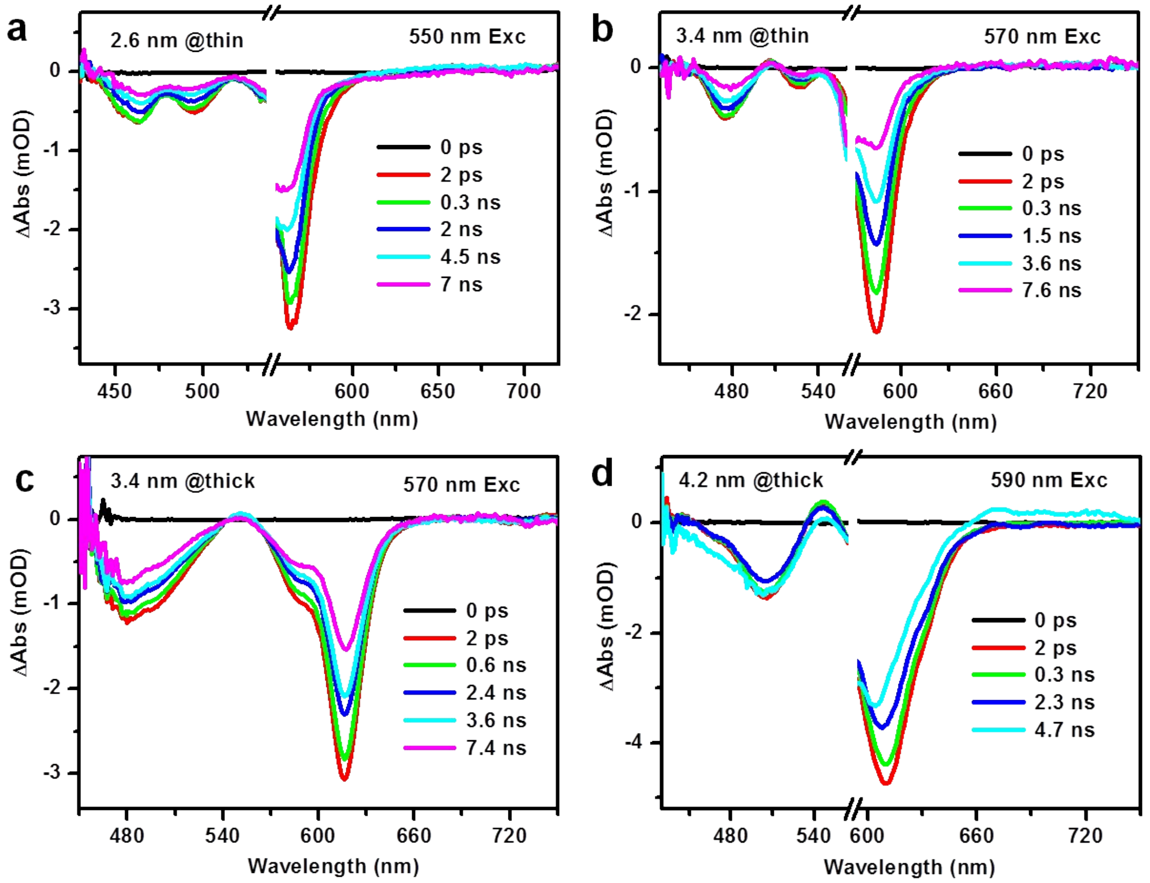


**Fig. S5** TA spectra of (a) 2.6 nm@thin DIRs, (b) 3.4 nm@thin DIRs, (c) 3.4 nm@thick DIRs, and (d) 4.2 nm@thick DIRs at indicated time delays. The excitation wavelengths are chosen to be slight on the blue side of the 1S exciton peaks of the cores and are indicated in the figures.

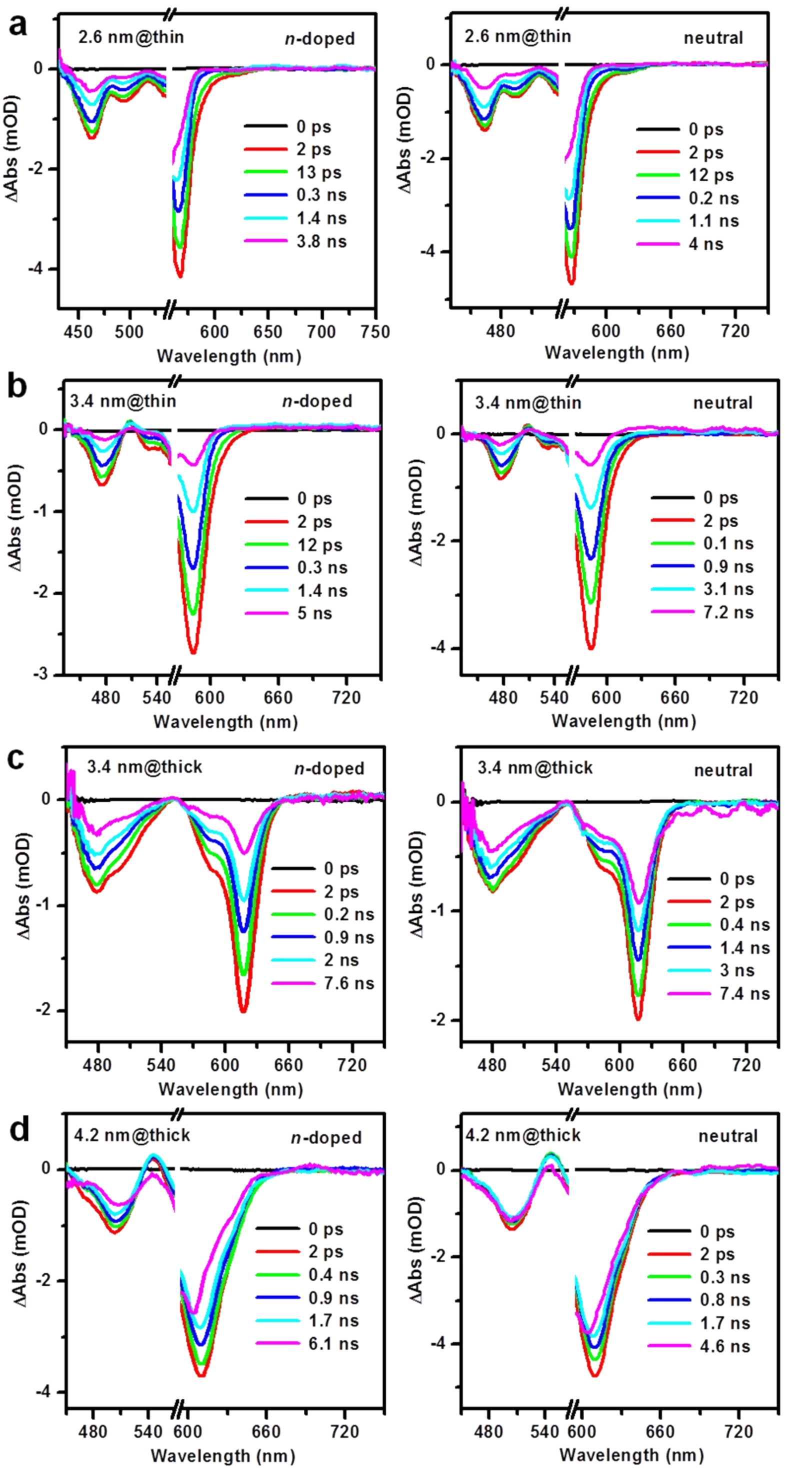


**Fig. S6** TA spectra of *n*-doped (left panels) and neutralized (by exposure to the air; right panels) samples for (a) 2.6 nm@thin DIRs, (b) 3.4 nm@thin DIRs, (c) 3.4 nm@thick DIRs, and (d) 4.2 nm@thick DIRs at indicated time delays. The excitation wavelengths are the same as those used in Fig. S5.

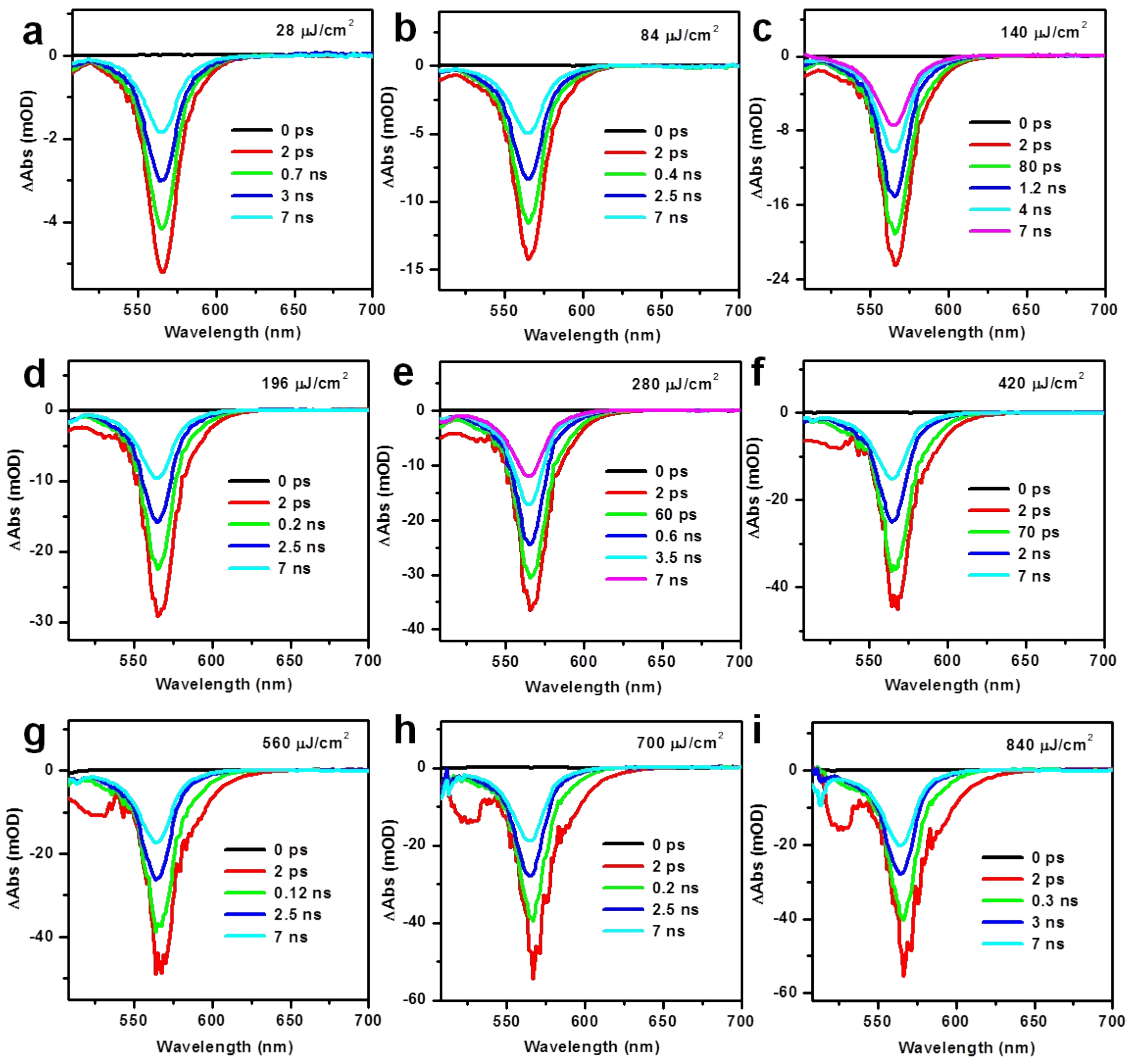


**Fig. S7** TA spectra of 2.6 nm@thin DIRs at indicated time delays following the excitation by 500 nm pulses with various energy densities. The per-pulse energy densities are indicated in the figure.

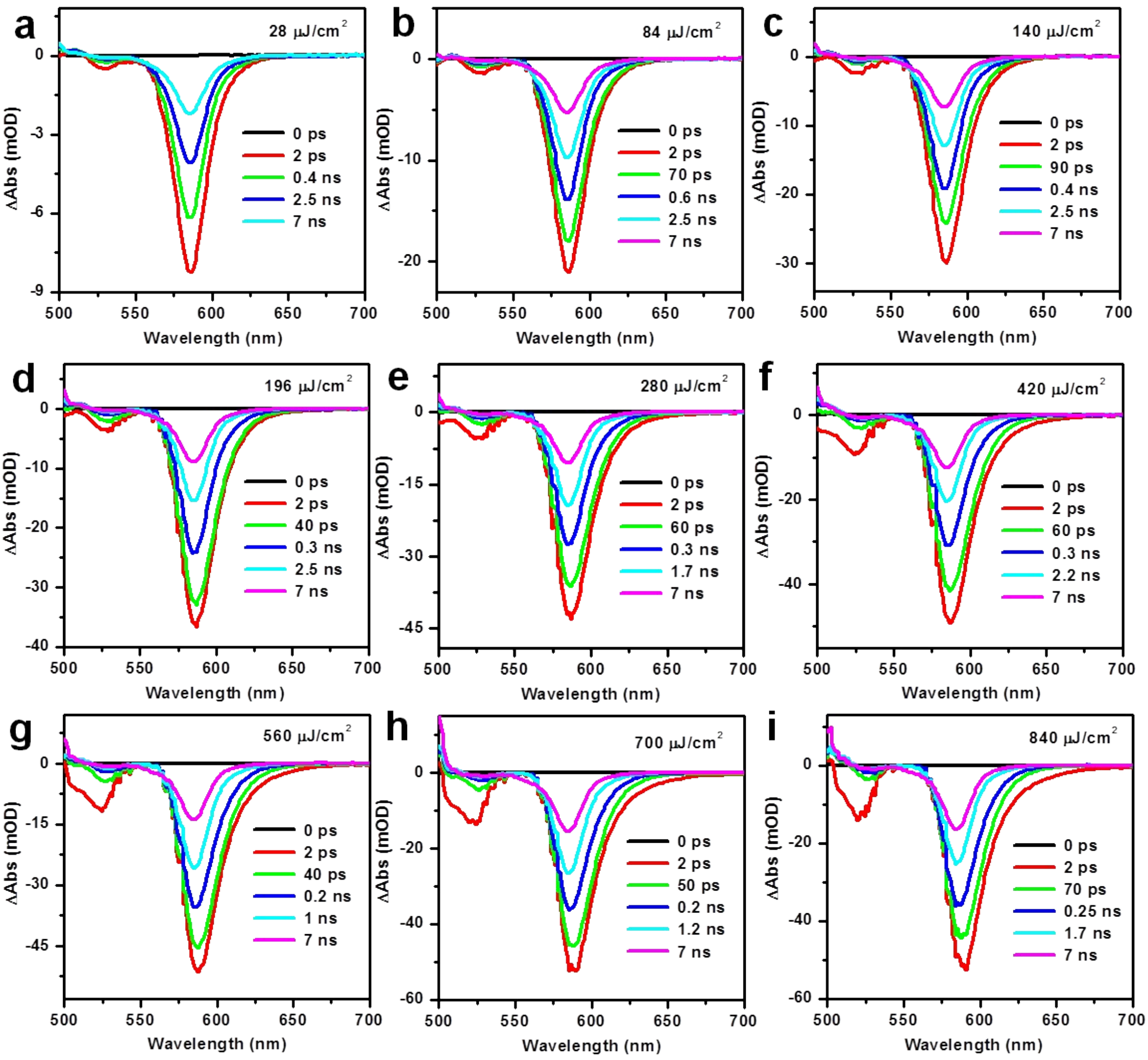


**Fig. S8** TA spectra of 3.4 nm@thin DIRs at indicated time delays following the excitation by 500 nm pulses with various energy densities. The per-pulse energy densities are indicated in the figure.

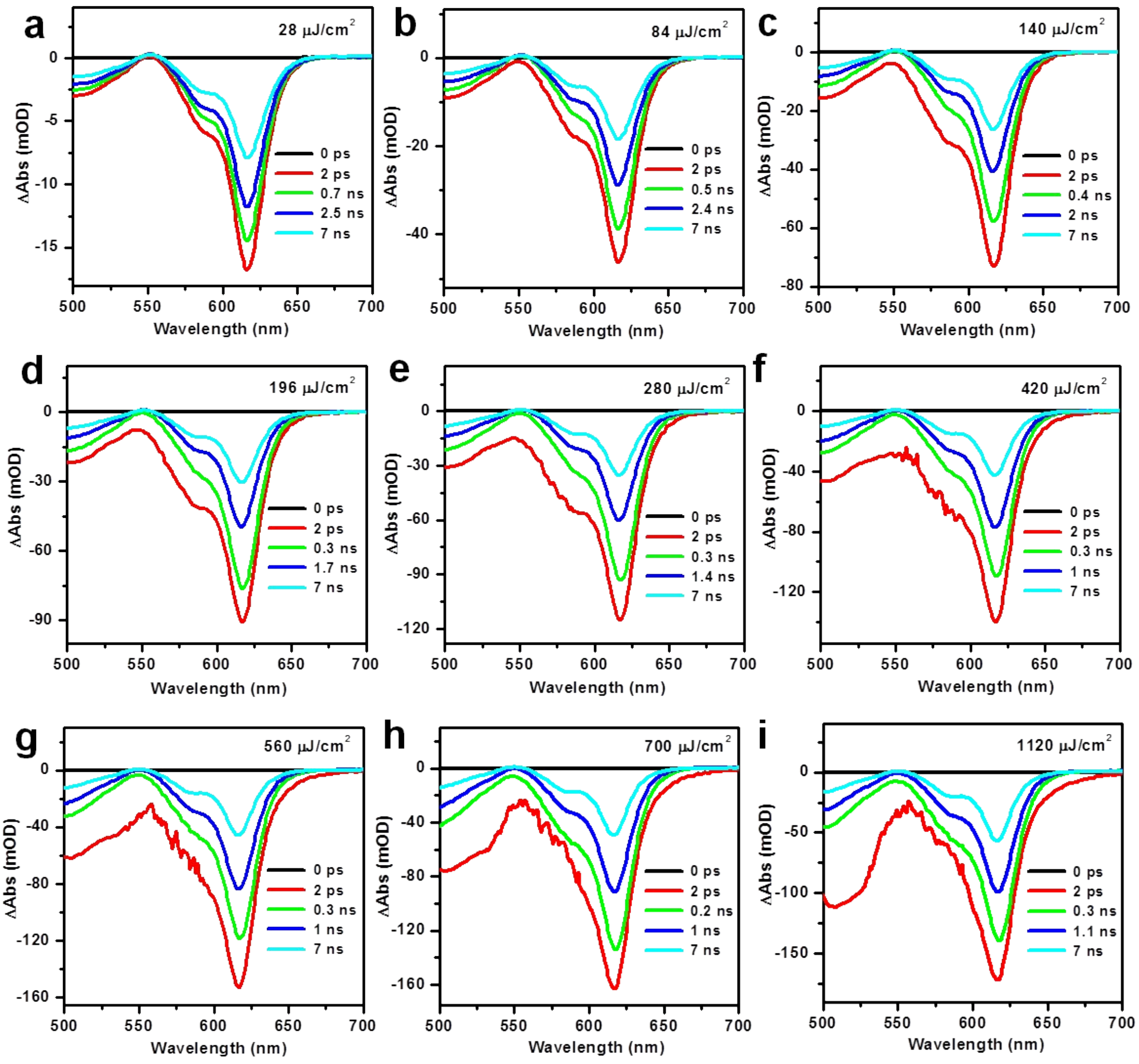


**Fig. S9** TA spectra of 3.4 nm@thick DIRs at indicated time delays following the excitation by 500 nm pulses with various energy densities. The per-pulse energy densities are indicated in the figure.

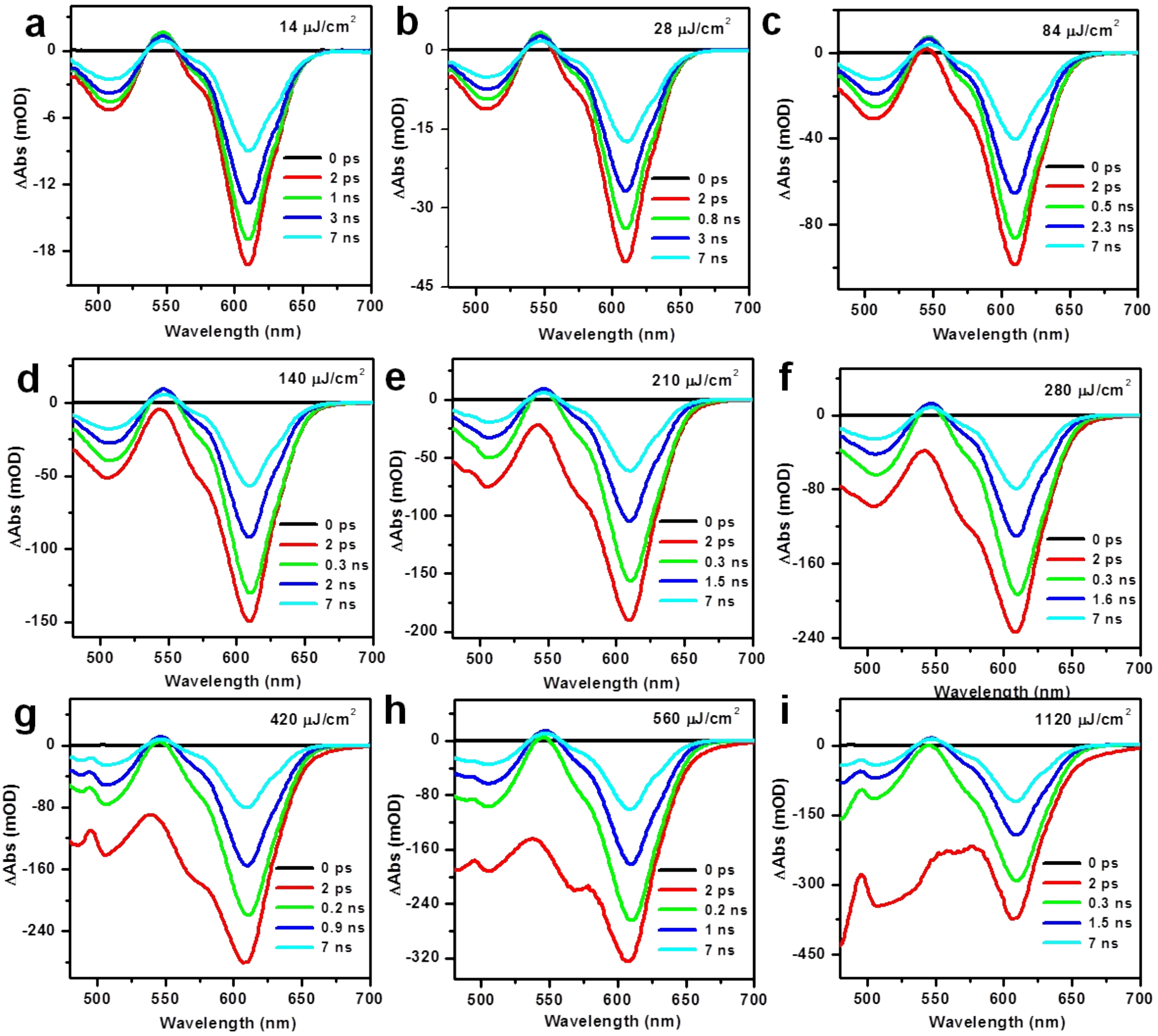


**Fig. S10** TA spectra of 4.2 nm@thick DIRs at indicated time delays following the excitation by 500 nm pulses with various energy densities. The per-pulse energy densities are indicated in the figure.

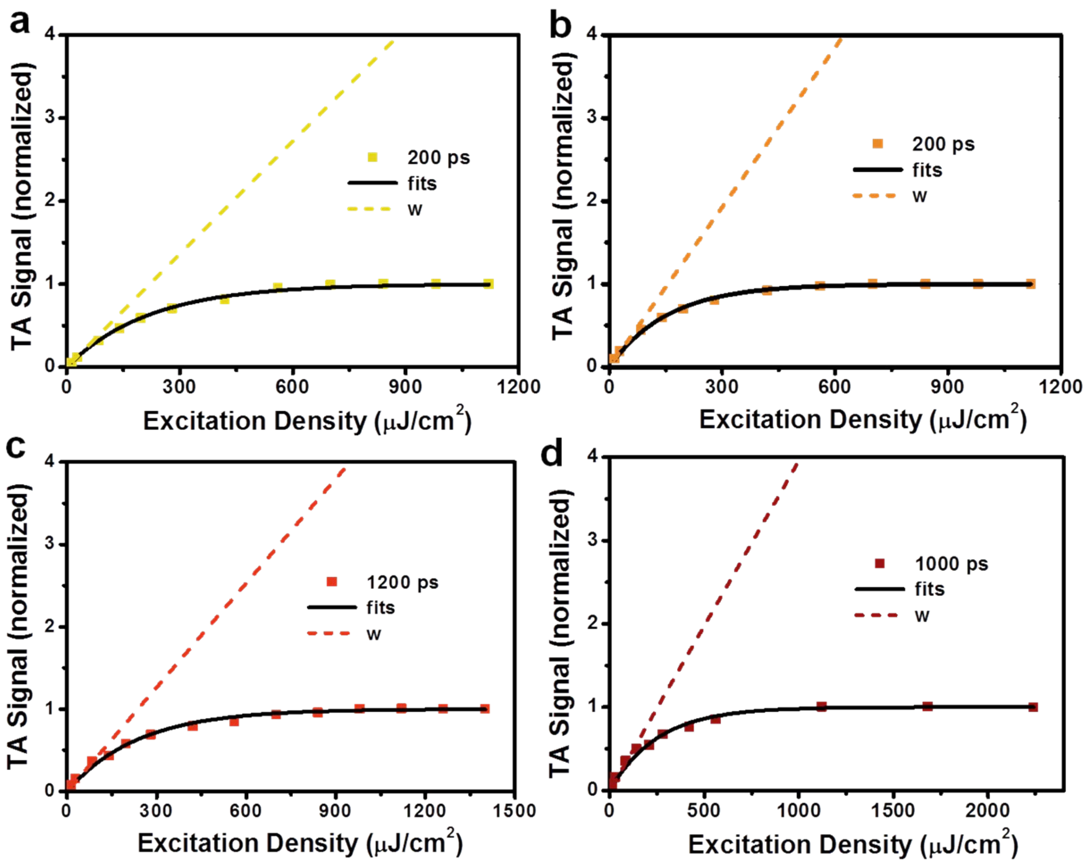


**Fig. S11** Plots of the rescaled signal amplitudes at the indicated times as a function of the excitation densities (colored squares) and their fits to a Poisson statistics model (black solid lins) for (a) 2.6 nm@thin DIRs, (b) 3.4 nm@thin DIRs, (c) 3.4 nm@thick DIRs, and (d) 4.2 nm@thick DIRs. According to this model, the statistics for the absorption of photons with energy much higher the band-gap is a Poissonian,[1] and the TA signals at delay times when Auger recombination have finished can be expressed as: $I \propto 1 - P(0) = 1 - e^{-\langle N \rangle}$, where $\langle N \rangle$ is the average number of photons absorbed per QD or the average exciton number per QD prior to Auger recombination. $\langle N \rangle$ is proportional to the pump laser fluence: $\langle N \rangle = Cj$. Therefore, the rescaled TA signals in the figures can be fitted with: $I \propto 1 - e^{-Cj}$, with $C$ as the only fitting parameter. Using the fitted $C$ value, the average exciton number $\langle N \rangle$ at each excitation energy density can be calculated (colored dashed lines).

**Synthesis of CdSe@CdS DIRs**

CdSe@CdS DIRs with various dimensions were synthesized using literature methods.[2-3] Wurtzite CdSe QDs of three different sizes were used as seeds for the epitaxy of CdS rods. Briefly, for synthesizing CdSe@CdS DIRs, 0.06 g cadmium oxide (CdO), 3 g tri-n-octylphosphine oxide (TOPO), 0.29 g octadecylphosphonic acid (ODPA), and 0.08 g hexylphosphonic acid (HPA) were degassed under vacuum for 1 hour at 150 °C. After heating to 350 °C under argon for half an hour, the mixture turned into clear solution, indicating the dissolution of CdO. At this point, 1.8 mL TOP was injected into the solution. In a separated container, sulfur injection solution (0.12 g S in 1.8 mL TOP) was mixed with CdSe QDs. The amount of seeds in a typical synthesis was $8\times10^{-8}$ mol. When the temperature of the Cd-containing solution was stabilized at 350 °C, the seed-containing Sulfur injection solution was quickly injected. The solution was allowed to maintain for different time to grow CdSe@CdS DIRs with various dimensions. The products were precipitated out of the reaction crudes by addition of ethanol. The precipitation processes were repeated for several times. Final DIRs were dispersed in toluene.

**Photochemical electron-doping of DIRs**

The photochemical doping experiments followed a literature method.[4] Briefly, $LiEt_3BH$ (1 M solution in tetrahydrofuran) was diluted to 0.01 M with toluene and was transferred together with DIRs in toluene into a glove box filled with $N_2$ atmosphere (oxygen level <0.1 ppm). The $LiEt_3BH$ solution was added dropwise into the DIR solution under vigorous stirring, which was then illuminated by a UV lamp to accelerate the reaction until it reached an equilibrated doped state. The degree of electron-doping was controlled by varying the

amount of $LiEt_3BH$ added and was monitored by UV-vis absorption spectra. The doped samples were sealed in custom-made airtight cuvettes (optical path 1 mm) and transferred out of the glove box for all optical measurements. The neutralized samples were obtained by exposing the doped samples to the air for a few minutes.

**Transient absorption (TA) measurements**

Femtosecond TA measurements were based on a regenerative amplified Ti:sapphire laser system (Coherent; 800 nm, 35 fs, 6 mJ/pulse, and 1 kHz repetition rate) and a TA spectrometer (femto-TA100; Time-Tech Spectra LLC). Briefly, the 800 nm output pulse from the amplifier was split in two parts. One part was used to pump a TOPAS Optical Parametric Amplifier (OPA) which generated wavelength-tunable pump beams. The other part was further split into two beams. One beam was attenuated with an N.D. filter and was focused onto a 2-mm-thick sapphire window to generate a white light continuum (WLC) as the probe beam. The probe beam was focused with an Al parabolic mirror onto the sample. After the sample, it was collimated and then focused into a fiber-coupled spectrometer with CMOS sensors and detected at a frequency of 1 KHz. The intensity of the pump pulse used in the experiment was controlled by N.D. filters. The delay between the pump and probe pulses was controlled by a motorized delay stage. The pump pulses were chopped by a synchronized chopper at 500 Hz. Samples were placed in 1 mm cuvettes and were vigorously stirred during all the measurements.

**Calculation of lifetimes and emission yields of multi-carrier states.**

According to the scaling laws established in the literature,[5] the radiative lifetime of biexcitons ($\tau_{XX,r}$) is related to the radiative lifetime of single excitons ($\tau_X$) via:

$$\tau_{XX,r} = \tau_X/4 \quad \text{(S1).}$$

The radiative lifetimes of trions can be expressed as:

$$\tau_{X*,r} = \tau_X/\beta \quad \text{(S2),}$$

where $\beta$ is the emission-rate enhancement factor of trions (X*) compared to neutral excitons. It has been shown that in monocomponent QDs with mirror symmetric conduction and valence bands, $\beta$ = 2. For quasi-type-II CdSe@CdS systems the repulsion between two delocalized electrons in $X^-$ reduces $\beta$ to ~1.7, whereas $\beta$ is still close to 2 for the positive trion as both holes remain confined to the core region, as in the neutral exciton.

The lifetime of biexcitons is contributed by its radiative ($\tau_{XX,r}$) and Auger ($\tau_{XX,A}$) lifetimes via:

$$1/\tau_{XX} = 1/\tau_{XX,r} + 1/\tau_{XX,A} \quad \text{(S3).}$$

The same applies to positive and negative trions. The Auger lifetimes of biexcitons and trions follow a superposition relationship:[6-7]

$$1/\tau_{XX,A} = 2/\tau_{X+,A} + 2/\tau_{X-,A} \quad \text{(S4).}$$

All the lifetimes for biexcitons and trions can be calculated using eqs. S1-S4, which are summarized in Table S1. The emission yields of biexcitons ($\Phi_{XX}$) or trions ($\Phi_{X*}$), relative to single excitons, are calculated using:

$$\Phi_{XX} = \tau_{XX}/\tau_{XX,r} \quad \text{(S5),}$$

$$\Phi_{X*} = \tau_{X*}/\tau_{X*,r} \quad \text{(S6).}$$

**Table S1. Lifetimes and emission yields of multi-carrier states in all DIR samples.**

| Sample | States | $\tau$ (ps) | $\tau_r$ (ps) | $\tau_A$ (ps) | $\Phi_r$[a] |
|---|---|---|---|---|---|
| 2.6 nm@thin | X | 8130 | 8130 | NA | 100% |

| | | | | | |
|---|---|---|---|---|---|
| | $X^-$ | 870 | 4782 | 1063 | 18.2% |
| | $X^+$ | 122 | 4065 | 126 | 3.0% |
| | XX | 57.8 | 2032 | 59.5 | 2.8% |
| 3.4 nm@thin | X | 8030 | 8030 | NA | 100% |
| | $X^-$ | 663 | 4726 | 771 | 14.0% |
| | $X^+$ | 188 | 4015 | 198 | 4.7% |
| | XX | 84.0 | 2008 | 87.7 | 4.2% |
| 3.4 nm@thick | X | 12900 | 12900 | NA | 100% |
| | $X^-$ | 1369 | 7588 | 1670 | 18.0% |
| | $X^+$ | 740 | 6450 | 836 | 11.5% |
| | XX | 303 | 3225 | 334 | 9.4% |
| 4.2 nm@thick | X | 12100 | 12100 | NA | 100% |
| | $X^-$ | 1713 | 7118 | 2256 | 24.1% |
| | $X^+$ | 762 | 6050 | 871 | 12.6% |
| | XX | 326 | 3025 | 365 | 10.8% |

a. Emission yields relative to single excitons

## References:


1. Klimov, V. I.; Mikhailovsky, A. A.; McBranch, D. W.; Leatherdale, C. A.; Bawendi, M. G. Quantization of multiparticle Auger rates in semiconductor quantum dots. *Science* **2000,** *287*, 1011-1013.
2. Carbone, L.; Nobile, C.; De Giorgi, M.; Sala, F. D.; Morello, G.; Pompa, P.; Hytch, M.; Snoeck, E.; Fiore, A.; Franchini, I. R.; Nadasan, M.; Silvestre, A. F.; Chiodo, L.; Kudera, S.; Cingolani, R.; Krahne, R.; Manna, L. Synthesis and Micrometer-Scale Assembly of Colloidal CdSe/CdS Nanorods Prepared by a Seeded Growth Approach. *Nano Lett.* **2007,** *7*, 2942-2950.
3. Wu, K.; Hill, L. J.; Chen, J.; McBride, J. R.; Pavlopolous, N. G.; Richey, N. E.; Pyun, J.; Lian, T. Universal Length Dependence of Rod-to-Seed Exciton Localization Efficiency in Type I and Quasi-Type II CdSe@CdS Nanorods. *ACS Nano* **2015,** *9*, 4591-4599.
4. Rinehart, J. D.; Schimpf, A. M.; Weaver, A. L.; Cohn, A. W.; Gamelin, D. R. Photochemical Electronic Doping of Colloidal CdSe Nanocrystals. *J. Am. Chem. Soc.* **2013,** *135*, 18782-18785.
5. Klimov, V. I. Multicarrier Interactions in Semiconductor Nanocrystals in Relation to the Phenomena of Auger Recombination and Carrier Multiplication. *Annual Review of Condensed Matter Physics* **2014,** *5*, 285-316.
6. Park, Y.-S.; Bae, W. K.; Pietryga, J. M.; Klimov, V. I. Auger Recombination of Biexcitons and Negative and Positive Trions in Individual Quantum Dots. *ACS Nano* **2014,** *8*, 7288-7296.

7. Wu, K.; Lim, J.; Klimov, V. I. Superposition Principle in Auger Recombination of Charged and Neutral Multicarrier States in Semiconductor Quantum Dots. *ACS Nano* **2017,** *11*, 8437-8447.
